# Developing Peer‐Mentored Cognitive Behavioural Therapy for Adolescents With Mild‐To‐Borderline Intellectual Disability and Anxiety: A Qualitative Study

**DOI:** 10.1111/jar.70297

**Published:** 2026-09-03

**Authors:** Iris Langereis, Jasmin Rahemenia, Renske D. Pols, Marieke G. N. Bos, Anika Bexkens

**Affiliations:** ^1^ GGZ Delfland Delft the Netherlands; ^2^ Leiden University Leiden the Netherlands

## Abstract

**Background:**

Youth with intellectual disabilities are at increased risk of developing anxiety disorders. Adapted CBT shows potential for youth with intellectual disabilities, but evidence for effectiveness is limited and mixed, urging the need for further optimisation. This study aims to develop a peer‐mentored CBT intervention, together with and for youth with mild‐to‐borderline intellectual disability and anxiety, based on scientific evidence, clinical expertise and patient preferences.

**Methods:**

In the intervention development phase, 40 stakeholders (youth with intellectual disability, parents, therapists, experts by experience) took part in focus groups and interviews, providing insight into clinical expertise and patient preferences.

**Results:**

Stakeholders expected peer‐mentored CBT to result in anxiety reduction and increased self‐esteem, due to maximised exposure and inclusion of a peer‐mentor. Stakeholders identified barriers and provided their views on solutions.

**Conclusions:**

Stakeholders see potential in peer‐mentored CBT. Including youth with intellectual disability was feasible and led to important insights for intervention development.

## Lay Summary

- Peer‐mentored CBT is a cognitive behavioural therapy (CBT) intervention designed together with and for youth with mild‐to‐borderline intellectual disability and anxiety.
- Youth with mild‐to‐borderline intellectual disability, parents, experts by experience and clinicians see positive potential in peer‐mentored CBT.
- Youth with mild‐to‐borderline intellectual disability, parents, experts by experience and clinicians agree that doing more in vivo, in‐session exposure is important to reduce anxiety. This is in line with previous literature.
- Youth with mild‐to‐borderline intellectual disability, parents, experts by experience and clinicians agree that including a peer‐mentor in anxiety therapy can be helpful. They also see that certain conditions related to feasibility and (social) safety are necessary for the peer‐mentor to participate successfully.
- When developing mental health care interventions, including youth with mild‐to‐borderline intellectual disability in research is feasible, desirable and important to provide patient perspectives.

## 1 Introduction

Anxiety disorders are one of the most prevalent psychiatric disorders in modern society (WHO 2004). Youth with intellectual disability are at increased risk of developing anxiety disorders (Einfeld et al. 2011; Green et al. 2015) with estimated prevalence rates ranging between 3% and 34% (Buckley et al. 2020; Mazza et al. 2020; Reardon et al. 2015). Cognitive Behavioural Therapy (CBT) is the first‐choice treatment for anxiety disorders (NICE 2014). Research suggests that people with intellectual disabilities can benefit from psychological treatments, such as CBT (Osugo and Cooper 2016), with most evidence for treating anger and depression (Graser et al. 2022; Tapp et al. 2023; Vereenooghe and Langdon 2013). For anxiety, less research has been done and the quality of the research varies strongly, with many uncontrolled trials and small sample sizes (Graser et al. 2022; Tapp et al. 2023; Unwin et al. 2016). Findings are mixed, with some studies (Vereenooghe and Langdon 2013; Unwin et al. 2016) reporting emerging evidence for CBT in people with (mild) intellectual disabilities and anxiety, while others (Graser et al. 2022; Tapp et al. 2023) refrain from drawing conclusions on effectiveness and state that when uncontrolled trials are excluded, no significant effect size is found.

Historically, it has been proposed that cognitive deficits in people with intellectual disabilities hindered engagement in traditional CBT (Bütz et al. 2000; Taylor et al. 2008). However, it has been shown that people with intellectual disabilities, mainly those with mild intellectual disabilities, have some of the skills required for CBT and that these skills can be taught (Bruce et al. 2010; Joyce et al. 2006; Taylor et al. 2008; Vereenooghe et al. 2015, 2016). For youth with intellectual disabilities, recent studies show that CBT is promising when therapy is adapted to their needs by matching cognitive, social and adaptive levels of functioning (Fynn et al. 2023; Hronis et al. 2017). Studies in this field are, however, still very scarce and effect sizes of effectiveness of CBT for anxiety treatment, adapted for youth with intellectual disabilities, are lower (Fynn et al. 2023) compared to youth without intellectual disabilities (Crowe and McKay 2017; Higa‐McMillan et al. 2016; Weisz et al. 2017). This urges the need to further optimise CBT for anxiety in youth with intellectual disabilities.

Guidelines for evidence‐based practice in psychology state that scientific evidence should be integrated with clinical expertise and patient perspectives in clinical decision‐making for individual patients, to provide the best possible care (APA Presidential Task Force on Evidence‐Based Practice 2006; American Psychological Association 2021). These three sources of information have increasingly also been incorporated in intervention development. There is a growing movement to involve patients' experiences in efforts to improve or redesign mental health services (Bate and Robert 2006; Boivin et al. 2014; Bombard et al. 2018; Carman et al. 2013; Richards et al. 2015) and studies increasingly show that including stakeholders' perspectives in shaping interventions leads to improved effectiveness, efficiency, quality of health services, self‐esteem and feelings of empowerment (for a review, see Bombard et al. 2018). Yet, youth with intellectual disabilities are often overlooked in research where patient input is included, despite that studies show that including youth with intellectual and/or developmental disabilities to share their perspectives is feasible when shaping interventions (e.g., Maenhout et al. 2023; Schwartz et al. 2020; Schwartz, Davern, et al. 2025; Schwartz, Vallabhaneni, et al. 2025). Therefore, the aim of the current study is to develop a new CBT intervention for the treatment of anxiety, together with and for youth with mild‐to‐borderline intellectual disability (defined as IQ between 50 and 85 and significant limitations in adaptive functioning, American Psychiatric Association 2013) and anxiety, based on scientific evidence, clinical expertise and patient preferences.

Previous studies have adapted CBT for anxious youth with mild‐to‐borderline intellectual disability by making changes to the delivery of CBT, including slowing the pace during sessions, increasing the time spent on practice and rehearsing, shortening session length and using visual support (Fynn et al. 2023; Hronis et al. 2017). These changes in delivery are no fundamental adaptations to the mechanisms of CBT and the way of learning and the process through which therapists support learning skills and acquiring insights remains unchanged (Dagnan et al. 2023). Such adaptations are considered important to improve the effectiveness of CBT for youth with intellectual disabilities. However, given the inconclusive evidence for the effectiveness of CBT, even with these changes incorporated, we believe that more is needed to boost effectiveness even further. Building on this work, peer‐mentored CBT was developed, based on two pillars: maximalisation of in vivo, in‐session exposure and the inclusion of a peer‐mentor.

First, a recent meta‐analysis on anxiety treatment in youth (not specifically youth with intellectual disabilities) suggests that in vivo in‐session exposure (i.e., facing a feared stimulus in real life during a therapy session to learn the feared outcome does not occur) is the driving factor behind CBT treatment success (Whiteside et al. 2020). Moreover, in vivo in‐session exposure therapy is expected to be an especially good fit for youth with mild‐to‐borderline intellectual disability, as studies investigating adaptations to treatment protocols for youth with intellectual disabilities emphasise the importance of ‘learning by doing’ (Hronis et al. 2017). ‘Learning by doing’ refers to reality‐based learning processes, such as hands‐on activities, role playing and practicing such that the individual experiences the learning process firsthand. These aspects are part of the mechanism of learning in exposure therapy and we therefore conclude that in vivo in‐session exposure should be a cornerstone of an intervention for youth with mild‐to‐borderline intellectual disability and anxiety.

Second, we aimed to identify a pathway which would be specifically applicable in treatment for youth with mild‐to‐borderline intellectual disability. Here, developmental neuroscience and behavioural studies point to social learning as a port‐of‐entry for intervention development that may be powerful specifically for adolescents with mild‐to‐borderline intellectual disability. Adolescence is a period characterised by increased sensitivity to social contexts and peer feedback (see Albert et al. 2013; Blakemore and Mills 2014; Do et al. 2019; Foulkes and Blakemore 2016; Giletta et al. 2021; Knoll et al. 2015; Somerville 2013; Steinberg and Monahan 2007), specifically in youth with intellectual disabilities (for a review, see Bexkens and Müller 2021). Social learning through peer observation might therefore be a pathway to enhance therapy by means of peer‐mentoring. Peer‐mentoring (defined here as a peer with lived experience acting as mentor for one who is new to those lived experiences) has become increasingly recognised as an intervention in Dutch and international mental healthcare services (GGZ Standaard 2024; Commonwealth of Australia 2013; Cyr et al. 2016; Department of Health (DH) 2012; US Department of Health & Human Services 2020). The inclusion of peers in therapy has been linked to improved self‐esteem in patients and increased self‐efficacy, personal growth and a feeling of worth and purpose in participating peers (Miyamoto and Sono 2012; Tisdale et al. 2021). It has also shown promising results regarding feasibility and acceptability of healthcare, personal growth, improved self‐esteem, social skills, emotional skills and quality of life in youth with intellectual and/or developmental disabilities (Athamanah et al. 2020; Curtin et al. 2016; Schwartz and Levin 2021; Schwartz, Davern, et al. 2025). Given the sensitivity to the social (peer) context in youth with intellectual disabilities, including a peer‐mentor to enhance social learning might be a powerful way to boost therapy effectiveness for youth with mild‐to‐borderline intellectual disability.

**FIGURE 1:**
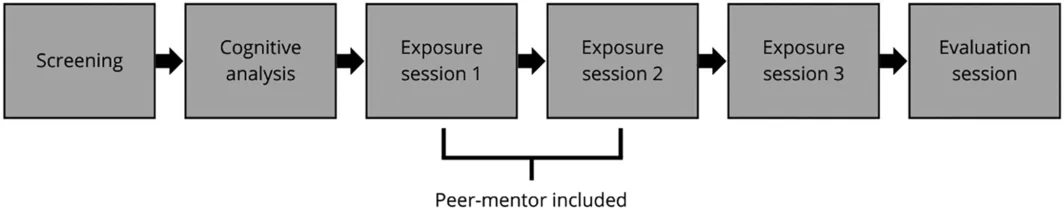
Visual overview of the prototype peer‐mentored CBT before the evaluation with stakeholders.

Based on scientific literature, we developed ‘peer‐mentored CBT’, a CBT intervention for youth with mild‐to‐borderline intellectual disability and anxiety. The intervention was initially developed by a team of researchers who largely also work in clinical practice (including, but not restricted to, the authors of this paper). It was designed for adolescents from 12 to about 21 years old who are diagnosed with any type of anxiety disorder that can be treated by means of in vivo exposure therapy. This includes, but is not limited to, situation or stimulus related anxiety disorders such as for example social anxiety disorder (prevalence in youth with intellectual disabilities of 5.4% (95% CI of 1.9%–14.6%), Maïano et al. 2018) or specific phobias (prevalence in youth with intellectual disabilities of 19.8% (95% CI of 11.8%–31.3%), Maïano et al. 2018). Cornerstones of peer‐mentored CBT are maximalisation of in vivo, in‐session exposure and inclusion of a peer‐mentor for modelling and observational learning. The peer‐mentor is a peer with lived experience with mild‐to‐borderline intellectual disability and anxiety treatment, including exposure. The intervention consists of a cognitive analysis session, three weekly exposure sessions of 3 h each and an evaluation session. In the cognitive analysis session, the fears are thoroughly analysed together with a therapist, inspired by other intensive exposure‐focused cognitive behavioural interventions (Davis 3rd et al. 2019; Öst and Ollendick 2017; Remmerswaal et al. 2021). Next, the patient takes part in three exposure sessions, guided by a therapist. The length of the sessions was derived from the empirically supported One‐Session Treatment model (OST; Davis 3rd et al. 2012). We tripled the number of sessions to provide sufficient repetition and slowing of pace. The peer‐mentor is included in the first two exposure sessions. In the evaluation session, the effect of the intervention and potential follow‐up care are discussed. A visual overview of peer‐mentored CBT is presented in Figure 1 and it is more thoroughly described in Appendix A. In line with our aim to develop an evidence‐based intervention, this study examined stakeholders' views on peer‐mentored CBT and how these can be incorporated into the intervention, by means of focus groups and interviews with stakeholders in these areas: patients, parents, therapists and experts by experience on mild‐to‐borderline intellectual disability.

## 2 Methods

### 2.1 Participants

Forty stakeholders participated in the study, which were youth with mild‐to‐borderline intellectual disability and mental health conditions, parents of youth with mild‐to‐borderline intellectual disability, therapists (i.e., (post‐master certified) psychologists, (post‐master certified) family and educational psychologists and psychiatrists) with experience in working with youth with mild‐to‐borderline intellectual disability (years of experience ranged from 5 to 35 years, M = 18.08 and SD = 9.49) and experts by experience with mild‐to‐borderline intellectual disability and mental health conditions (see Table 1). Experts by experience were young adult peer‐mentors with mild‐to‐borderline intellectual disability and lived experience with mental health conditions, who have learned to share their experiences professionally through training (from now on referred to as ‘experts by experience’). Given that no younger age group trained as experts by experience was available, the group of experts by experience consisted of young adults. Inclusion criteria varied per group. Inclusion criteria for youth were: (1) mild‐to‐borderline intellectual disability, (2) age between 12 and 21 years old and (3) experience with psychotherapy. Parents could participate if they had a child who met these criteria, participation of this child in the focus groups was not required for a parent to participate. Therapists could participate if they had experience in working with youth with mild‐to‐borderline intellectual disability in mental health care, there were no requirements for the number of years of experience. For the experts by experience, there were no additional inclusion criteria other than their above‐described job description. There were no specific age requirements for this group. There were no exclusion criteria for participation. Focus groups were organised by stakeholder type. Stakeholders were recruited via word of mouth, posters and flyers, telephone recruitment and via posts on social media. Stakeholders were recruited at various mental health care institutions in the Netherlands.

**TABLE 1:** Demographic data of the participating stakeholders.

Group	*N*	Birth sex (*N* male)^a^	Age	
			M	SD
Therapists^b^	15	2	44.00	8.86
Youth	9	2	17.67	2.65
Parents	8	4	51.62	7.07
Adult experts by experience	8	4	28.75	6.63

^a^ Participants only reported to be male or female as birth sex and no participant reported a different gender identity than their birth sex.

^b^ Of all 15 therapists, 13 shared their demographic data, which are displayed here.

### 2.2 Procedures

This study was approved by the Leiden University Psychology Research Ethics Committee under proposal number Bexkens‐V1‐4143. Informed consent was signed by all participants. For youth below 16 years, youngsters provided informed assent and their parent(s)/caregiver(s) signed consent. Youth 16 years and older signed informed consent themselves, in line with Dutch regulations. Yet, parents of all youth were informed about participation via a separate information letter. The consent forms were adapted to promote readability and understandability for individuals with mild‐to‐borderline intellectual disability, by means of shortening sentences, simplifying language use and including pictures. Participants received compensation in the form of a €10 gift card. Focus groups were led by two researchers: one researcher led the conversation by addressing the topics and the other researcher monitored the time and the group process. Four researchers were included in data collection, indicated by their initials: A. B. (female, 40 years old, principal investigator and licensed clinical psychologist), I. L. (female, 27 years old, PhD candidate and psychologist), R. P. (female, 40 years old, licensed psychologist‐in‐training to become a licensed clinical psychologist) and S. N. (female, 26 years old, student assistant). Focus groups had a planned duration of 90 min, which in practice varied from 45 min (youth focus groups, for feasibility reasons such as limiting fatigue and mental load) to 90 min. Focus groups and interviews for youth and experts by experience were adapted to meet the needs of individuals with mild‐to‐borderline intellectual disability. The focus groups and interviews were held at a location known to the participants, with a familiar counsellor present to support the participants. Language used was simplified and short, active sentences were used. To include sufficient individuals per stakeholder group, focus groups were supplemented with individual interviews (which had a duration of 45–60 min) with three parents and one youth. These were participants who were unable to attend on the set dates of the focus groups, but who did want to participate. Focus groups/interviews for therapists and parents took place via Zoom videoconference or face to face, focus groups/interviews for youth and adult experience experts took place face to face. Focus groups/interviews were audio and video recorded. Data was collected via a semi‐structured approach, outlined in Box 1. This approach included an open discussion about general best practices in treatment for youth with mild‐to‐borderline intellectual disability, followed by a discussion of the topics described in Box 1. All components and topics described in Box 1 were addressed in all focus groups and interviews. Participants were prompted to elaborate their answer when needed. Topics were discussed in varying order, depending on the course of the discussion.

BOX 1 **Contents of the focus groups and interviews, including a topic list.**

**Table:** 

Introductions and explanation: the researchers introduce themselves and explain the goal of the session, the rules of the focus group (e.g. do not interrupt each other, be respectful to each other) and the agenda for the session. Thereafter the participants introduced themselves. (10 minutes) Group discussion (prompted by open questions): what are general best practices in treatment for youth with mild‐to‐borderline intellectual disability? (15 minutes) Introduction of peer‐mentored CBT by a researcher, presenting the idea via a PowerPoint presentation. (5 minutes) Group discussion: evaluation of peer‐mentored CBT by means of topic list (30 minutes): Topic list: What are your first impressions? Do you think peer‐mentored CBT will lead to anxiety reduction? What are advantages and disadvantages to peer‐mentored CBT? What kind of person should the peer‐mentor be? And how/based on what can we match the peer‐mentor to the patient? What are requirements for the therapist providing peer‐mentored CBT? What is important in the collaboration between the therapist and the peer‐mentor? What is important in the collaboration between the patient and the peer‐mentor? What does the peer‐mentor need to be supported in their role? What does the therapist need to be supported? *Additional questions for therapists:* Is doing extensive diagnostic assessment beforehand required for being able to provide peer‐mentored CBT properly? Do you expect an effect of peer‐mentored CBT on self‐efficacy? Closing. (5 minutes)

### 2.3 Data Processing and Analyses

Audio‐records were transcribed using the online tool ‘Amberscript’ and were checked for accuracy with the video‐records by master students. We used thematic analysis to analyse the data (Braun and Clarke 2006). Transcripts were qualitatively coded using the online tool ‘Atlas.ti’ by three researchers (A.B., I.L. and J.R. (female, 30 years old, research assistant and psychologist)), who performed cross coding to ensure sufficient overlap in coding. Beforehand, we established a set of deductive codes based on literature on social learning theory and best practices in working with youth with mild‐to‐borderline intellectual disability (see Box 2). During coding, these were supplemented with inductive data‐driven codes (presented in the Supporting Informations S1). Inductive codes were created in consensus with all coding researchers in frequent meetings. Coded transcripts were checked to see whether newly created codes would also apply to previously coded excerpts. When all transcripts were coded, a final list of codes was printed and a first thematic map was constructed. Themes were identified at a semantic, explicit level (Boyatzis 1998) and were constructed based on coded excerpts with shared overarching meaning. The thematic map was reviewed and final alterations were made, after which the final set of themes was communicated with all stakeholders to perform a member check. The member check consisted of an overview of the final thematic map with a description of our preliminary findings. It was performed via e‐mail for therapists and parents and for youth and experts by experience it was communicated via their therapist or counsellor (in face‐to‐face contact), to promote accessibility. Stakeholders were asked whether they recognized their views in the themes described. Stakeholders did not report disagreement.

BOX 2 **Deductive code set.**

**Table:** 

*Social learning based codes* Social learning (general) Observational learning Modelling Identification Attention Retention Motivation Reproduction Reinforcement	*Codes based on best practices in therapy for youth with mild‐to‐borderline intellectual disability* Learning environment Structure and predictability Social network and generalisation Communication and language Social‐emotional profile

## 3 Results

Fifteen themes emerged from the data, which are presented in Box 3. Themes were grouped into five overarching topics: (i) result of the intervention, (ii) characteristics of the sessions in peer‐mentored CBT, (iii) social learning mechanisms in peer‐mentored CBT, (iv) the peer‐mentor in peer‐mentored CBT and (v) the learning environment in peer‐mentored CBT.

BOX 3 **Thematic map per topic.**

**Table:** 

**Topic (i): Result of the intervention** Theme included: 1. Result of the intervention	
**Topic (ii): Characteristics of the sessions**. Themes included: 2. Characteristics of the session Subtheme: active elements 3. Mapping the anxiety complaints 4. General evaluation of peer‐mentored CBT Subtheme: suggestions	**Topic (iii): Social learning mechanisms**. Themes included: 5. Social learning mechanisms 6. Mediational processes
**Topic (iv): The peer‐mentor** Themes included: 7. Characteristics and competencies of the peer‐mentor 8. Role of the peer‐mentor 9. Inter relational aspects Subtheme: collaboration between patient and peer‐mentor 10. Support for the peer mentor 4. General evaluation of peer‐mentored CBT Subtheme: (dis)advantages for the peer‐mentor	**Topic (v): The learning environment** Themes included: 11. Learning environment Subtheme: broad learning environment 12. Characteristics of the patient 13. Characteristics of the therapist 9. Inter relational aspects Subtheme: collaboration between therapist and peer‐mentor 14. Therapist skills 15. Feasibility for the therapist

### 3.1 Topic (i): Result of the Intervention

All stakeholder groups expected peer‐mentored CBT to result in anxiety symptom reduction and increased self‐efficacy of the patient. Parents and therapists also expected increased self‐efficacy in peer‐mentors as a result of participation, due to feelings of competence, helping and contributing to mental health growth of a peer. As stated by a therapist*:* ‘To teach is to learn twice’.

### 3.2 Topic (ii): Characteristics of the Sessions in Peer‐Mentored CBT

All stakeholder groups deemed maximalising in vivo in‐session exposure important to increase treatment effectiveness. All stakeholders underlined the added value of ‘doing instead of only talking’ in therapy, especially for youth with mild‐to‐borderline intellectual disability as it relies less on analysing cognitive processes. All stakeholder groups expected doing in‐session exposure (as opposed to via homework assignments) to enhance chances of treatment success, as illustrated by this youngster: ‘Usually in therapy, I discuss what I can practice at home and when I have to actually do it, it doesn't always work out. Because I have to do it all by myself and then I might end up not doing it at all’. Youth, parents and therapists described that including psychoeducation and explaining the rationale of treatment is important to increase motivation. Experts by experience, parents and therapists described that incorporating the daily lives of patients in therapy (i.e., real‐life, personal examples) can enhance treatment compliance, by making the exercises directly relevant and applicable for patients. Therapists suggested to incorporate a homework period for stronger consolidation of treatment effect.

Concerning practical aspects, all stakeholder groups questioned whether three sessions would be enough to obtain sufficient treatment effect and expected the duration of the sessions (3 h) might be too long due to mental fatigue and limited sustained attention common in youth with mild‐to‐borderline intellectual disability. Experts by experience and therapists advised including breaks in the sessions. Benefits of 3‐h‐long sessions were also described by all stakeholder groups, such as sufficient time to settle down, less appointments and more progress per session. Therapists discussed that the aetiology of anxiety symptoms should guide the decision whether to apply peer‐mentored CBT. Therapists discussed that anxiety symptoms in youth with mild‐to‐borderline intellectual disability often reflect underlying factors such as trauma or stress, for example when anxiety emerges because one feels they cannot meet the expectations of the environment in terms of cognitive and adaptive abilities. In these situations, therapists deemed alternative forms of treatment or guidance as more appropriate. However, several therapists emphasised that exposure can still play an important role in treatment when anxiety symptoms are present, as exposure may be necessary to effectively address the anxiety itself. Therapists advised to only implement additional diagnostic assessment when necessary for treatment, for example to determine if peer‐mentored CBT is feasible and suits the individual needs of a patient.

### 3.3 Topic (iii): Social Learning Mechanisms in Peer‐Mentored CBT

Youth and therapists described observational learning and modelling as key mechanisms for treatment success, especially for youth with mild‐to‐borderline intellectual disability. Therapists described that the demonstration of ‘brave behaviour’ provides the patient with a visual example of what is expected, thereby increasing the chance of success in replicating the modelled behaviour. Therapists expected the effectiveness of modelling to be even stronger if the model would also experience some fear while taking part in exposure exercises, as stated by a therapist: ‘If the peer‐mentor experiences some anxiety, the peer‐mentor will model being brave and show that it is okay to find something scary and do it anyway’. All stakeholder groups expected that the effect of observational learning would be larger when modelling is performed by the peer‐mentor (compared to a therapist), due to a feeling of identification. One parent stated: ‘It is really about recognizing and acknowledging and the feeling of not being alone. I think that is a real power of this intervention’. All stakeholder groups expected that behaviour modelled and feedback given by the peer‐mentor would be experienced by the patient as more credible and authentic. All stakeholder groups marked sharing lived experience with the patient as a core element of being a peer‐mentor. Shared experiences could refer to similar anxieties or a similar therapy process. Youth, parents and therapists expected self‐disclosure by the peer‐mentor about their own fears and treatment process to strengthen the feeling of identification, to increase normalisation of having fears and to increase feelings of equality. All stakeholder groups described that a ‘click’ between the patient and peer‐mentor, based on informal or personal aspects such as mutual interest, would be important for identification. Hence, all stakeholder groups described the necessity of an informal introductory session for the patient and peer‐mentor to ‘get acquainted’ before the start of treatment. All stakeholder groups saw value in a deliberate matching procedure based on age, where youth, experts by experience and parents expressed expected value in additional matching based on informal similarities such as mutual interests.

Concerning positive reinforcement, therapists and parents described their experience that youth with mild‐to‐borderline intellectual disability are especially sensitive to positive rewards. They therefore expected this to be an effective component of peer‐mentored CBT. Therapists furthermore expected social reinforcement to occur when the patient is able to perform the feared behaviour while having a peer as a spectator. However, some parents were concerned about youth engaging in behaviour they do not feel comfortable with because they might feel pressured, due to expected susceptibility to peer influence in youth with mild‐to‐borderline intellectual disability. Also, parents and therapists shared concerns about patient dependency, worrying that a patient may come to rely too much on the (presence of the) peer‐mentor and therefore not learn to trust their own capacities, for which it was described important to also practice in the absence of the peer‐mentor. Lastly, youth, parents and therapists suggested that the therapist should also engage in positive reinforcement towards the peer‐mentor, to promote feelings of self‐efficacy.

### 3.4 Topic (iv): The Peer‐Mentor in Peer‐Mentored CBT

All stakeholder groups agreed that being a peer‐mentor may not be for everyone: it would require certain characteristics, competencies and prerequisites. Concerning peer‐mentor characteristics, all stakeholder groups agreed that the patient and peer‐mentor should be matched based on age. Parents and therapists pointed out that mental or social–emotional age should also be considered, to provide the best fit on being a ‘peer’. On the desired level of intellectual and adaptive functioning for a peer‐mentor, stakeholder groups differed in their opinion. Some youngsters expressed a desire for a peer‐mentor with mild‐to‐borderline intellectual disability due to perceived mutual understanding and acceptance; other youngsters expressed they would rather be supported by someone who might potentially be more skilled compared to themselves. Parents, experts by experience and some therapists argued that a peer‐mentor of similar intellectual and adaptive functioning would be the best match because of the expected perceived identification. However, some therapists argued that skills they deemed essential for the role of peer‐mentor might be less prevalent in peer‐mentors with mild‐to‐borderline intellectual disability.

Concerning peer‐mentoring competencies, all stakeholder groups agreed that engaging in positive reinforcement is a key element of the peer‐mentor role. Modelling was described by therapists as another key element, both in line with the above‐described social learning mechanisms. Youth mentioned a peer‐mentor should be skilled at expressing understanding and acceptance. Parents mentioned a peer‐mentor should be good at listening to the patient and be able to take another person's perspective (i.e., mentalising). Therapist also deemed mentalising an important competency for a peer‐mentor, because they deemed it important to keep the focus of the intervention on the patient. They expressed concern that in their experience, mentalising is often compromised in youth with mild‐to‐borderline intellectual disability, potentially hindering the ability of youth with mild‐to‐borderline intellectual disability to fulfil a peer‐mentor role.

Stakeholders discussed potential pitfalls of peer‐mentor participation. To tackle these, stakeholders suggested certain prerequisites. Youth, parents and therapists mentioned that peer‐mentors may re‐experience their anxieties after modelling the exposure exercise and/or witnessing fearful patients. One youngster illustrated: ‘If I were a peer‐mentor and I just recovered from my anxiety disorder, and I were to help someone with the same kind of anxiety, I would find that tricky to help someone else and having to dive into that anxiety again’. Youth, parents, and therapists considered sufficient distance to past anxiety complaints an important prerequisite to reduce the chance of the peer‐mentor re‐experiencing their anxieties. Youth furthermore suggested to include peer‐mentors who have experience with similar anxiety therapy but different anxiety complaints, to tackle this issue. Therapists suggested the absence of significant comorbid complaints, such as severe depressive symptoms or suicidal tendencies, as another prerequisite. Furthermore, parents and therapists expected peer‐mentors might lack certain (therapeutic) skills, for which experts by experience, parents, therapists and some youth (but not all) agreed that providing a peer‐mentor training program would be of added value and many deemed including a peer‐mentor training to be a prerequisite for successful peer‐mentor participation. These stakeholders described the training should for example include clarification of expectations of the peer‐mentor role, teaching helpful peer‐mentoring behaviour and teaching basic communicative skills through for example practicing and role‐playing.

### 3.5 Topic (v): The Learning Environment in Peer‐Mentored CBT

All stakeholder groups emphasised the importance of a safe learning environment, adapted to the needs of youth with mild‐to‐borderline intellectual disability. All stakeholder groups described the importance of taking into account the appropriate adaptive, cognitive and social emotional level of a patient. Youth and experts by experience suggested adjusting communication and language by using short and simple sentences and adjusting the pace to patients' individual needs. Therapists and parents suggested using visual support, such as pictograms, to emphasise certain therapeutic aspects and to help memory consolidation. All stakeholder groups agreed that sessions should be well‐structured and predictable. Experts by experience, parents and therapists expressed that therapists providing peer‐mentored CBT should have received training in CBT, training in working with mild‐to‐borderline intellectual disability youth and preferably also already gained experience in working with youth with mild‐to‐borderline intellectual disability. All stakeholder groups agreed that the feeling of being able to fall back upon a therapist who can guide and support, would be important in supporting the peer‐mentor. Youth and experts by experience mentioned being able to discuss their role pre‐ and post‐session as supportive for peer‐mentors. Therapists mentioned that peer‐mentors, if they would also have mild‐to‐borderline intellectual disability, might require support in practical and organisational aspects of coming to appointments, such as setting reminders and discussing the route to the appointment. Experts by experience, parents, therapists and youth suggested to incorporate these aspects into a peer‐mentor training. Therapists described that properly training peer‐mentors would also reduce the workload for therapists providing peer‐mentored CBT. Therapists furthermore described that therapists providing peer‐mentored CBT would require facilitation from the organisation (i.e., being provided enough time to prepare for sessions, meetings with colleagues to discuss cases, being enabled to do diagnostics), a clear protocol and specific training in peer‐mentored CBT to enhance feasibility for therapists.

Experts by experience, parents and therapists underlined the importance of involving parents (or the broader network) in the intervention. Therapists described that generalisation to the home environment can be challenging when parents are not involved. They underlined the importance of teaching parents how they can assist in breaking with avoidance behaviours related to the anxiety and to tolerate distress in their child. Parents suggested that parents should be included in the intervention through individual parental guidance sessions. Youth were less conclusive about parental involvement: some saw added value in including parents; others did not as much. Youth and experts by experience expressed a desire for autonomy and some parents also described that a balance should be struck between sufficient parental involvement versus maintaining their child's feelings of autonomy in the therapeutic process.

The topic of autonomy was discussed more in depth. Mainly youth and experts by experience expressed their concerns about the amount of information shared between professionals, deeming it important that professionals communicate with, instead of about, patients. Youth and experts by experience expressed the importance of feeling autonomous by having a say during therapy. As described by a youngster: ‘If I am enabled to have a say or choice, I will feel more comfortable and I will likely open up more’. Therapists described that peer‐mentors should be informed about certain aspects of the patient to prepare for their role, without oversharing. Therapists and parents expected that providing too much information about the patient to the peer‐mentor might elicit a hierarchical relationship between patient and peer‐mentor, reducing feelings of identification and hindering treatment success. Therapists suggested sharing the information in‐session, together with the patient. For the intervention to be successful, stakeholders concluded that the relationship between therapist and peer‐mentor should be based on trust, such that the peer‐mentor knows that they can fall back on the therapist when needed, but that they also receive some autonomy when interacting with the patient. One therapist described it as: ‘The therapist and peer‐mentor can work together like Batman and Robin’.

## 4 Discussion

The aim of the current study was to develop a new CBT intervention for the treatment of anxiety, together with and for youth with mild‐to‐borderline intellectual disability and anxiety, based on scientific evidence, clinical expertise and patient preferences. Overall, stakeholders had a positive attitude towards peer‐mentored CBT and expected peer‐mentored CBT to result in anxiety symptom reduction and increased self‐efficacy in patients and peer‐mentors. Aat the same time, stakeholders had important suggestions for improvement. All stakeholder groups expected the combination of maximalisation of in vivo in‐session exposure and including peer‐mentors to be effective and fitting for youth with mild‐to‐borderline intellectual disability. Scientific literature about in vivo in‐session exposure (Whiteside et al. 2020) support this claim, as well as scientific literature about the inclusion of peers in therapy for youth with intellectual and/or developmental disabilities (Athamanah et al. 2020; Curtin et al. 2016; Schwartz and Levin 2021) and without disabilities (De Beer et al. 2022; Gopalan et al. 2017). In line with earlier studies (De Beer et al. 2022; Miyamoto and Sono 2012; Schwartz et al. 2020; Schwartz, Davern, et al. 2025; Schwartz, Vallabhaneni, et al. 2025), self‐disclosure was described as eliciting feelings of trust and equality, increasing identification and strengthening treatment effect. Modelling and positive (social) reinforcement were described as key elements and especially fitting for youth with intellectual disability, in line with research about best practice in therapy for youth with intellectual disabilities (Hronis et al. 2017; Witt and Vinter 2013).

This study provided a starting point for further optimisation of peer‐mentored CBT. Stakeholders shared concerns with the current form of peer‐mentored CBT and provided suggestions for change. We evaluated which suggestions to incorporate based on whether they were often expressed and whether they were also supported by scientific literature. A full overview of the changes to peer‐mentored CBT is included in Appendix B. First, youth, parents and therapists shared concerns that the peer‐mentor would re‐experience anxiety complaints during sessions. Some therapists described this as desirable because they expected this would strengthen identification and provide an example of how to cope with anxiety during exposure exercises. However, therapists expected this to only hold when the degree of experienced anxiety would be manageable for the peer‐mentor. Therefore, stakeholders described sufficient distance to own past anxiety complaints as an important prerequisite. Youth suggested including peer‐mentors with similar anxiety therapy experience but different anxiety complaints. Literature about peer support in youth without intellectual disabilities describes that the full absence of past symptoms is not required for successful peer supporting, but the capacity to self‐reflect about recovery, understand what happened and having moved on, is required (for a review, see De Beer et al. 2022). Difficulties in social–emotional cognitive functioning in youth with intellectual disabilities might complicate the ability to self‐reflect and grow distance to past anxiety complaints. In line with the suggestions by youth stakeholders, we will include peer‐mentors with similar therapy experiences but different (past) anxiety complaints. The estimation about whether a youngster has grown sufficient distance to their complaints (i.e., whether past anxiety complaints have been sufficiently treated such that participation as peer‐mentor is feasible and can be done safely) needs additionally be assessed for each potential peer‐mentor individually and in consultation with the youngster, their parents and if possible the involved professional(s), before participation. Absence of significant current comorbid complaints was also suggested by therapists as a prerequisite for peer‐mentors. These criteria were incorporated as screening criteria for peer‐mentors. A peer‐mentor training was proposed by all stakeholder groups to overcome these and several other concerns. When including peers in therapy, providing a training is common (Gopalan et al. 2017) and earlier stakeholder‐driven research also underlined the importance that mentors with intellectual and/or developmental disabilities have potential to deliver interventions but may require training in certain skills and content areas of the intervention (Schwartz et al. 2020; Schwartz and Hwang 2022; Schwartz, Vallabhaneni, et al. 2025). More specifically, Schwartz, Vallabhaneni, et al. (2025) showed that training supported fidelity of intervention delivery and increased self‐confidence in peer‐mentors with intellectual and/or developmental disabilities. Hence, a peer‐mentor training is now included in the study protocol. The peer‐mentor training was designed in cooperation with an expert by experience with mild‐to‐borderline intellectual functioning, to enhance acceptability and in line with earlier studies (Schwartz, Vallabhaneni, et al. 2025). During the peer‐mentor training, peer‐mentors receive psychoeducation on anxiety and guidance regarding expected peer‐mentor behaviours. They also develop a plan on how to support the patient and engage in discussions about maintaining appropriate professional distance, strategies to preserve this distance and approaches to managing any anxieties they may experience while participating.

Second, stakeholders suggested to include the rationale of treatment, psychoeducation about anxiety and using daily‐live examples of patients, which are all key elements to CBT (Grills et al. 2023; King and Boswell 2019). Studies show that these key elements are important for promoting positive outcome expectancies, treatment credibility, normalisation, alliance‐building and engagement (Constantino et al. 2018; Grills et al. 2023). Using daily‐live examples has furthermore been related to enhanced learning in literature about youth with intellectual and/or developmental disabilities (D'Amico et al. 2005; Sauter et al. 2009). These aspects are therefore now included in peer‐mentored CBT. Third, therapists suggested including a homework period after the exposure phase. Research into adult anxiety disorder therapy shows an added effect of doing homework on anxiety therapy effectiveness (Mausbach et al. 2010). Research into homework in youth anxiety therapy however yields mixed results (Arendt et al. 2016; Hughes and Kendall 2007; Lee et al. 2019). In their recent review, Klein et al. (2024) suggest that homework quality may explain these mixed results and describe that increasing homework quality is key. A homework period (i.e., a period of 4 weeks after finishing the exposure sessions) was therefore included in peer‐mentored CBT. In the homework period, patients practice at home with facing their fears. The homework exercises are matched to the type of anxiety the patient experiences. Examples of homework exercises include interactions with feared animals or locations (for specific phobia or agoraphobia), to interact with peers or ask a question in class (for social phobia). The homework exercises are designed together with the patient and parent(s) in a separate session to increase chances of homework compliance, homework effectiveness and to increase feasibility and safety of practicing with facing fears.

Third, mainly therapists and parents expressed a desire for parental involvement. Research into including parents in therapy is inconclusive, as studies yield mixed results (Byrne et al. 2023). However, for youth with mild‐to‐borderline intellectual disability, the need to involve parents or a broader support system is important to enhance feasibility and generalizability (Hronis et al. 2017, 2022). Hence, peer‐mentored CBT was adapted such that parents are included in different phases of the intervention: the cognitive analysis session (final 15 min), the third exposure session (final 30 min), the homework pre‐session and in the homework period. Parents are not included in the majority of the exposure sessions to promote patient autonomy. This desire was strongly expressed by stakeholders, especially by youth and experts by experience and also in recent research (Westera et al. 2023). Furthermore, stakeholders described best practices to overcome common barriers in therapy with youth with mild‐to‐borderline intellectual disability, which included adaptations in language and delivery of interventions and in training of professionals. These adaptations were already incorporated in peer‐mentored CBT and in future studies into the feasibility, acceptability and effectiveness of peer‐mentored CBT, we aim to investigate whether the adaptations in peer‐mentored CBT sufficiently overcome these barriers. Last, therapists described that individual assessment based on aetiology of the anxiety complaints is needed to decide whether peer‐mentored CBT is the appropriate intervention. As peer‐mentored CBT was not designed for one type of anxiety disorder specifically, but rather for any type of anxiety that can be treated with in vivo exposure, this assessment is now included in the screening.

Not all insights from the current study were incorporated in peer‐mentored CBT. First, most stakeholders agreed that having a similar age (mental and/or chronological age), shared experiences, superficial features and common interests would strengthen identification. Studies that look into model‐observer similarity yield mixed results, with some studies showing a potential positive effect of similarity on learning (Braaksma et al. 2002; Hartmann et al. 2020; Kim 2007) while other studies do not find this effect (Groenendijk et al. 2013; Hoogerheide et al. 2016, 2017, 2018; Krämer et al. 2016). For example, sharing superficial features such as having the same gender as the model has been shown in previous studies to result in perceiving the model as more similar to oneself, but does not directly enhance learning (Hoogerheide et al. 2018; Krämer et al. 2016). Moreover, literature about peer inclusion in therapy shows that the effectiveness of including peers is related to feelings of equality, authenticity and empathy (De Beer et al. 2022), aspects which relate to similarities in age and experiences, but not as much to superficial features. Another recent study investigating the acceptability of a peer‐mentoring program for youth and young adults with intellectual and/or developmental disabilities describes that in their intervention, their initial intention was to match mentor and mentee based on (informal) similarities, but were mostly unable to due to lack of feasibility (Schwartz, Davern, et al. 2025). These authors still observed a positive effect of the peer‐mentoring intervention, despite the inability to perform deliberate matching. Moreover, peer‐mentors included in their study described no such need for informal matching, deeming it more important to have overlap in life experiences with disability and/or mental health. Hence, deliberate matching based on informal similarities is not incorporated in peer‐mentored CBT. Similar (mental) age and similar anxieties or therapy process will remain the basis of matching. In matching based on age, there can be a challenge when mental age and chronological age are far apart. The feeling of being a ‘peer’ and having shared lived experiences may be more likely with an individual of a similar chronological age, but when there is a large gap between mental age and chronological age, this may not provide a good match. This is a consideration to be evaluated at the individual level each time when matching is performed.

**FIGURE 2:**
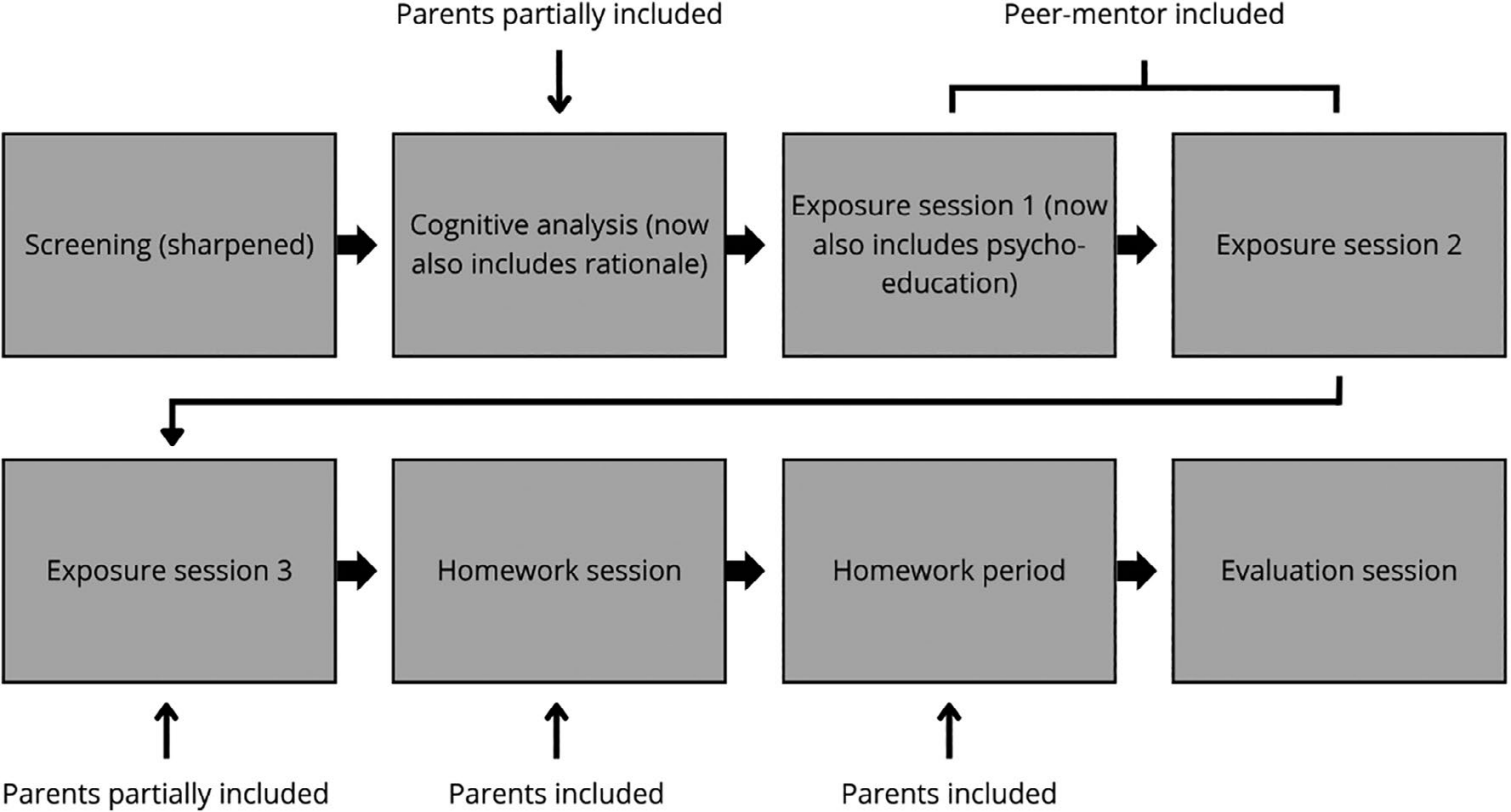
Visual overview of peer‐mentored CBT after the evaluation with stakeholders.

Second, although stakeholders differed in opinion on whether to include peer‐mentors with mild‐to‐borderline intellectual disability, previous studies suggest including peer‐mentors with intellectual disabilities is feasible (Schwartz and Levin 2021). Also, by including a peer‐mentor training specifically targeting potential difficulties for youth with mild‐to‐borderline intellectual disability, feasibility is expected to increase (Schwartz, Vallabhaneni, et al. 2025). Therefore, having mild‐to‐borderline intellectual disability has become an inclusion criterium for becoming a peer‐mentor in peer‐mentored CBT. Third, stakeholders shared doubts about the length and number of the sessions, expecting three sessions to be too few and 3 h to be too long. They, however, also expected that sufficient breaks in between would suffice to promote feasibility. Youth with intellectual and/or developmental disabilities often take longer to complete tasks (Hartman et al. 2010), which 3‐h‐long sessions can facilitate. As 3‐h‐long exposure sessions are also feasible for children as young as four years old (Davis 3rd et al. 2019), peer‐mentored CBT will first be pilot tested in the original form of three sessions of 3 h, with regular breaks and small, manageable tasks. A visual overview of peer‐mentored CBT after incorporation of the suggestions by stakeholders is presented in Figure 2 and it is more thoroughly described in Appendix B.

Like all studies, this study had strengths and limitations. The major strength of this study was the stakeholder‐driven approach. Views of stakeholders in the current study largely overlap with findings in previous studies, expanding the foundation for performing CBT as evidence‐based practice for youth with intellectual disabilities. Concerning limitations, a combination of focus groups and individual interviews was used due to recruitment difficulties. Although this form of triangulation is often used in qualitative research (Katz‐Buonincontro 2022; Lambert and Loiselle 2008) and no substantial differences were found between interviews and focus groups, the aspect of group discussion lacks in interviews. Youth stakeholders were recruited at only two different mental health care institutions and parents and experts by experience at only one institution, which provides a limited context for their experiences with mental health care. Therapists were recruited from a wide range of institutions; however, still wider recruitment of participants from more institutions would be informative for future research. Furthermore, data on the clinical experiences of patients and experts by experience were not consistently collected, nor was data collected on the background or clinical experience of parents. This knowledge could have provided a more thorough description of the viewpoint from which these stakeholders shared their experiences. Last, participants did not have direct experience in working with peers in therapy, so their evaluation and perspectives are based on their experiences with individual therapy and therefore speculative. When peer‐mentored CBT will be piloted, first‐hand experiences of stakeholders will be evaluated.

## 5 Conclusion

The aim of this study was to develop peer‐mentored CBT, a new intervention for the treatment of anxiety, together with and for youth with mild‐to‐borderline intellectual disability and anxiety, based on scientific evidence, clinical expertise and patient preferences. This paper describes the scientific evidence on which peer‐mentored CBT was developed and provides insights on patient preference and clinical expertise, which were incorporated into the intervention. Stakeholders saw positive potential in peer‐mentored CBT. Including youth with mild‐to‐borderline intellectual disability in research has been feasible and important and this group should not be overlooked in research or when shaping mental healthcare. Peer‐mentored CBT will continue to be developed and studied. The next step for peer‐mentored CBT is to be pilot tested for feasibility, acceptability and first signs of effectiveness.

## Author Contributions

A.B. developed the intervention and designed the current study together with colleagues. A.B., R.D.P. and I.L. were included in data collection and acquisition. A.B., I.L. and J.R., were included in data analysis and interpretation. A.B., I.L., R.D.P., J.R. and M.G.N.B. were included in writing and critically revising the manuscript. All authors approved of submission of the manuscript for publication and agreed to be accountable for all aspects of the work.

## Funding

This research was funded by a research grant granted to Prof. dr. Anika Bexkens by ZonMw (636320013). As the researchers have no personal benefit or gain from this research.

## Conflicts of Interest

The authors declare no conflicts of interest.

## Supporting information


**Supporting Information: S1.** Code book.

## Data Availability

Due to the sensitive nature of the data collected, the data are not shared publicly.

## Appendix A Visual Overview of the Contents of Peer‐Mentored CBT Before Inclusion of Patient Preferences and Clinical Expertise

Before the conduction of the current study, the prototype of peer‐mentored CBT was shaped based on the literature. Peer‐mentored CBT was inspired by other intensive exposure interventions (Davis 3rd et al. 2019; Öst and Ollendick 2017; Remmerswaal et al. 2021), which can be seen in the structure and contents of the intervention. In Figure 1 in the main text, it is depicted in its original form, before execution of this study.

### Screening

Patients are eligible for peer‐mentored CBT when they have mild‐to‐borderline intellectual and any form of anxiety for which exposure‐focused CBT would be indicated.

### Cognitive Analysis

As a starting point, the fears of the patient are analysed by means of a cognitive analysis session. This is a session of 60–90 min in which the fears are examined in a conversation between the therapist and the patient. The therapist aims to unravel the fears in detail, including antecedents, avoidance and safety behaviours. Exposure exercises are pinpointed to the exact fears, based on this analysis session.

### Exposure Sessions

Next, the patient takes part in three intensive, 3‐h‐long exposure sessions, of which the peer‐mentor participates in the first two. The time between the cognitive analysis and the first exposure session is 2 weeks and the time between the subsequent exposure sessions is 1 week. In these 3‐h‐long sessions, the therapist, patient and peer‐mentor (except for the third session) take part in intensive exposure therapy, in different settings and by means of different exercises. In the first session, the peer‐mentor mainly models the desired behaviour and the patient is allowed to watch most of the time. In the second session, this gradually shifts towards less modelling and more encouraging by the peer‐mentor and more to execution of the exercises by the patient. The therapist mainly supervises and guides the sessions. In the last session, the peer‐mentor is no longer included and the patient takes part in the exposure exercises by themselves.

### Evaluation Session

In a final, 45 to 60 min evaluation session, the patient, parents and therapist together discuss what the patient has learned and whether the treatment was sufficiently successful.

## Appendix B Overview of the Changes Made to the Prototype Peer‐Mentored CBT Intervention as a Result of This Study

As a result of this study, changes were made to the peer‐mentored CBT intervention. In Figure 2 in the main text, it is depicted in its altered form.

### Screening and Training

Prerequisites to participation for the peer‐mentor were sharpened based on input by stakeholders. Peer‐mentors can now only take part after receiving the peer‐mentor training. Inclusion criteria for peer‐mentors now include sufficient distance to past anxiety complaints, absence of severe co‐morbid psychiatry (i.e., severe depression, personality disorders, current trauma‐related complaints, suicidality or eating disorders) and absence of current personal stressors.

### Cognitive Analysis

Explaining the rationale of treatment is now included in the cognitive analysis session. Exercises are shaped to daily life examples of patients. Parents are now included in the final part of the cognitive analysis, such that the patient can first discuss the fears individually and parents can also add to this by sharing their views.

### Exposure Phase

Psycho‐education about anxiety and repeating the rationale is now included in the exposure sessions, with emphasis on the first session. During the first session, psycho‐education is included by having the peer‐mentor explain how they cope with their fears, what happens when they get anxious and how that anxiety passes. Also, while the peer‐mentor is modelling the behaviour, the therapist explains what is happening in conversation with the patient, while observing the peer‐mentor. The therapist also engages in psycho‐education in between exercises. The rationale of treatment is also repeated again and is also provided by the peer‐mentor by self‐disclosure about their own therapy process. Often breaks are now included, in which there is room for informal contact between the patient and peer‐mentor. Parents are now included in the last hour of the third exposure session, such that the patient has a spectator to watch them perform the brave behaviour and for parents to see what their child is capable of.

### Homework Session

A homework period of 4 weeks (see below) is now included in the intervention. To prepare for this period, a homework session is now included. In this 45–60‐min session, patient and parents are invited to shape homework exercises which are relevant and feasible. The therapist guides this process and provides suggestions. The final product is a homework exercise plan for 4 weeks which is shaped by the patient. In this session, a first short evaluation of the exposure phase of the intervention is done. Parents are included in the full session to promote generalizability to the home environment by providing tips and tools for guidance of the homework exercises.

### Homework Phase

A homework period of 4 weeks is now included in the intervention. In this period, the patients execute the homework plan. This plan should include exercises in multiple settings and on multiple occasions.

### Evaluation Session

In the evaluation session, the combined effect of the exposure phase and the homework phase is evaluated.
