# Supplementary material for: Developing Peer‐Mentored Cognitive Behavioural Therapy for Adolescents With Mild‐To‐Borderline Intellectual Disability and Anxiety: A Qualitative Study
Source: J Appl Res Intellect Disabil. 2026 Sep 3;39(5):e70297. doi: 10.1111/jar.70297 (PMC13541418; doi:10.1111/jar.70297)
Supplement: Supplementary file 1 [file JAR-39-e70297-s001.docx]

**Supplementary materials: Code book**

*Deductive codes*

| Code name | Description | Themes |
| --- | --- | --- |
| Learning environment | The environment/context in which therapy takes place and the aspects associated with it, such as the working relationship. | Learning environment |
| Cognitive profile | Adjustments in treatment based on cognitive abilities in cases of mild-to-borderline disability | Characteristics of the patient |
| Communication and language | Adjustments in treatment based on language and communication appropriate for mild-to-borderline disability | Therapist skills |
| Social network and generalization | Involving social networks in treatment for the purpose of generalization | Learning environment  (Subtheme: broad learning environment) |
| Structure and predictability | Adjustments in treatment based on needs in cases of mild-to-borderline disabilityin terms of structure and predictability, including clarity | Learning environment |
| Social emotional profile | Adjustments in treatment based on the social-emotional profile | Characteristics of the patient |
| Social learning | Umbrella term for matters that generally refer to social learning | Social learning mechanisms |
| Doing instead of only talking | The added value of being active during treatment compared to only having conversations | Learning environment |
| Modeling | Occurrence of behavior/actions (by peer) | Social learning mechanisms |
| Reinforcement | The process of encouraging or establishing a belief or pattern of behavior. Encouragement of the patient (by the peer) | Mediational processes |
| Identification | Extent to which the patient can identify with the peer | Social learning mechanisms |
| Observational learning | How the patient can learn by watching the peer do something (learning from modeling) | Social learning mechanisms |
| Attention | Paying attention to the behavior and its consequences and form a mental representation of the behavior. | Mediational processes |
| Retention | How well the behavior is remembered. | Mediational processes |
| Reproduction | This is the ability to perform the behavior that the model has just demonstrated. | Mediational processes |

*Inductive codes*

| Code name | Description | Themes |
| --- | --- | --- |
| Connecting to daily practice | During treatment, make connections to the patient's daily practice and recent events. | Characteristics of the session  (Subtheme: active elements) |
| Making mild-to-borderline disability-relevant adaptations to therapy | Adjustments that the therapist can (or cannot) make to the treatment so that it is more tailored to the mild-to-borderline disability target group | Therapist skills |
| Explaining rationale | Explaining to patients why certain exercises/treatments are being performed and what their purpose is | Characteristics of the session  (Subtheme: active elements) |
| Individualizing | Tailor treatment based on patient needs | Therapist skills |
| Treatment skills | Therapist's treatment skills in general (not specifically focused on mild-to-borderline disability) | Therapist skills |
| Explanation of complaints | Where does the fear come from? | Mapping the anxiety complaints |
| Diagnostics | Required diagnostics and added value of diagnostics prior to treatment for people with mild-to-borderline disability, focused on anxiety symptoms | Mapping the anxiety complaints |
| Therapeutic relationship | The relationship between the patient and the therapist. | Learning environment |
| Discrepancy social and cognitive skills | Taking into account varying abilities among people with mild-to-borderline disability | Characteristics of the patient |
| Experience in working with mild-to-borderline disability | The experience a therapist has in treating young people with mild-to-borderline disability | Therapist characteristics |
| Frame of reference therapist | Therapist's own frame of reference that colors their actions | Therapist characteristics |
| Education about mild-to-borderline disability | Training that a therapist has received specifically on mild-to-borderline disability | Therapist characteristics |
| Impact of work setting therapist | Impact of the work context of a therapist on their actions and thinking | Therapist characteristics |
| Working evidence-based | Working on the basis of evidence-based techniques or protocols | Therapist skills |
| Duration of sessions | The duration of the individual exposure sessions in peer-mentored CBT | Characteristics of the session |
| Generalization | The extent to which learned skills extend to the home situation | Learning environment  (Subtheme: broad learning environment) |
| Lived experience peer-mentor | Whether a peer has (had) the same fear as the patient | Characteristics of the peer-mentor |
| Who is the peer-mentor? | Characteristics of the peer-mentor | Characteristics of the peer-mentor |
| Distance to own complaints peer-mentor | The distance the peer-mentor has from their own issues. Also: the extent to which their own issues have been processed. | Characteristics of the peer-mentor |
| Matching | Matching the peer-mentor and patient based on various factors | Relationships |
| Group treatment | Suggestion to turn peer-mentored CBT into a group treatment | General evaluation of peer-mentored CBT  (Subtheme: suggestions) |
| Which anxieties? | For which fears is peer-mentored CBT applicable? | Mapping the anxiety complaints |
| Mentalizing ability peer-mentor | The extent to which the peer-mentor can empathize with the patient | Characteristics of the peer-mentor |
| Self-efficacy peer-mentor | Effect on participation as a buddy on feelings of self-efficacy in the peer-mentor | Result of the intervention |
| Risks for peer-mentor | Risks of having a peer-mentor participate in therapy | General evaluation of peer-mentored CBT  (Subtheme: (dis)advantages for the peer-mentor) |
| Peer group of the peer-mentor | The suggestion that peer-mentors can meet each other and receive guidance | Support for the peer-mentor |
| Added value of peer-mentor | Added value of having a peer-mentor participate | General evaluation of peer-mentored CBT |
| Role of the peer-mentor (general) | What is the role of the peer-mentor during treatment? | Role of the peer-mentor |
| Using supporting materials | Use supportive tools such as images, texts, visuals, etc., or strategies such as working memory exercises (tangle, etc.). | Learning environment |
| Self-disclosure peer-mentor | The extent to which the peer-mentor discusses their own issues with the patient and dares to be vulnerable | Characteristics of the peer-mentor |
| Training the peer-mentor | Passages mentioning the possibility/importance of training for peer-mentors | Support for the peer-mentor |
| Written protocol | Therapists' need for a manual for providing treatment | General evaluation of peer-mentored CBT  (Subtheme: suggestions) |
| Relationship between therapist and peer-mentor | The relationship between the therapist and the peer and what it must entail | Relationships |
| Getting acquainted | Introduction between the various individuals involved in treatment | Relationships |
| Professionality relationship peer and patient | The way in which patient and peer-mentor relate to each other in their relationship | Relationships |
| Alternating peer-mentors | Suggestion to rotate/alternate peer-mentors within a single treatment session | General evaluation of peer-mentored CBT  (Subtheme: suggestions) |
| Using virtual reality | Suggestion to use virtual reality in treatment | General evaluation of peer-mentored CBT  (Subtheme: suggestions) |
| Listening to the patient | The importance of listening carefully to the patient and showing understanding | Therapist skills |
| Patience | The importance of showing patience | Therapist skills |
| Openness in patient | The extent to which the patient can open up to the therapist or peer-mentor | Characteristics of the patient |
| ‘Click’ between patient and peer-mentor | A good connection/feeling between patient and peer-mentor and its importance for the successful outcome of treatment. | Relationships |
| Including an independent observer | An additional person for observing during the sessions | General evaluation of peer-mentored CBT  (Subtheme: suggestions) |
| Involving the system | Ideas about involving parents/relatives/broader system in treatment | (Subtheme: broad learning environment)  Learning environment |
| Therapeutic skills peer-mentor | The skills that peer-mentors need to provide effective support | Characteristics of the peer-mentor |
| Affinity in helping others peer-mentor | The extent to which the peer-mentor has an affinity for helping people | Characteristics of the peer-mentor |
| Age peer-mentor | How old the peer-mentor should be, relative to the patient | Characteristics of the peer-mentor |
| Number of sessions | The number of sessions required for treatment | Characteristics of the session |
| Information provision to peer-mentor | Providing the peer-mentor with information about the patient's treatment or complaints/characteristics | Support for the peer-mentor |
| Exposure therapy | Passages discussing the confrontation of fear or the implementation of exposure | Characteristics of the session  (Subtheme: active elements) |
| Safety- and avoidance behaviors | Passages in which avoidance (avoiding the feared situation) and safety behaviors (behaviors you use to reduce the fear or tension you experience at that moment, during the feared situation) are mentioned | Mapping the anxiety complaints |
| Alternative treatment methods | Treatment methods that are not covered by CBT and that are suggested | General evaluation of peer-mentored CBT  (Subtheme: suggestions) |
| Psycho-education | Passages about providing psychoeducation about anxiety to the patient, for example: what is anxiety, how does it work, specific information about the frightened thing | Characteristics of the session  (Subtheme: active elements) |
| Autonomy | Passages that mention the importance of autonomy in the patient, for example freedom of choice. Also physical autonomy | Therapist skills |
| Support for peer-mentors (general) | Umbrella code for what a peer-mentor needs to function properly as a peer-mentor | Support for the peer-mentor |
| Skills deficit | Passages in which the patient's lack of skills is identified as a factor contributing to anxiety | Mapping the anxiety complaints |
| Overestimation | Passages in which overburdening the patient is mentioned as a factor that plays a role in anxiety | Mapping the anxiety complaints |
| Advantages for peer-mentor | The benefits for the peer-mentor of participating as a peer-mentor in treatment | General evaluation of peer-mentored CBT  (Subtheme: (dis)advantages for the peer-mentor) |
| Arguments against participation of the peer-mentor | Passages describing arguments against allowing a peer-mentor to participate | General evaluation of peer-mentored CBT  (Subtheme: (dis)advantages for the peer-mentor) |
| Cognitive interventions | Passages describing the use or importance of cognitive interventions (not exposure) | Characteristics of the session  (Subtheme: active elements) |
| Screening | Determine in advance for which patients the treatment is appropriate. | Mapping the anxiety complaints |
| Safety | Passages concerning patient safety in terms of privacy, anonymity, and social safety | Therapist skills |
| Normalizing | Normalizing anxiety during treatment | Therapist skills |
| Opinion about effectiveness | Passage in which participants share their opinions on whether they think it will work or not | General evaluation of peer-mentored CBT |
| Intelligence profile peer-mentor | Ideas about the intelligence profile of the peer-mentor | Characteristics of the peer-mentor |
| Goal of the intervention | What is the purpose of peer-mentored CBT and the suggestion to specify this | General evaluation of peer-mentored CBT  (Subtheme: suggestions) |
| Teaching skills to patients | Teaching skills during treatment that could reduce symptoms | Therapist skills |
| Coordination between professionals | Passage mentioning the importance of coordination between colleagues for the treatment | Relationships |
| Feasibility for therapist (general) | Aspects that make treatment more feasible for the therapist | Feasibility for the therapist |
| Support for therapist | What support does the therapist need in order to carry out the treatment properly? | Feasibility for the therapist |
| Fixed peer-mentor group | Suggestion to work with a small group/pool of regular peer-mentors who help out more often | General evaluation of peer-mentored CBT  (Subtheme: suggestions) |
| Reward for peer-mentor | Will the peer-mentor be rewarded, and if so, how? Also suggestions for rewards | General evaluation of peer-mentored CBT  (Subtheme: suggestions) |
| Mentalizing ability peer-mentor | Passages about whether the peer-mentor has sufficient mental availability to actually have space within themselves to be able to act effectively as a peer-mentor. | Characteristics of the peer-mentor |
| Practical considerations | Practical considerations regarding peer-mentored CBT | General evaluation of peer-mentored CBT  (Subtheme: suggestions) |
| Sustaining factors | Factors perpetuating anxiety in the child and in their environment | Mapping the anxiety complaints |
| Treatment credibility | Passages about trust (or lack thereof) in treatment | General evaluation of peer-mentored CBT |
| Positive feedback/suggestions | What characteristics should a therapist have in order to provide treatment (in the broadest sense of the word)? | General evaluation of peer-mentored CBT |
| Characteristics of the therapist (general) | Passages that discuss the characteristics that a therapist should possess for treatment | Therapist characteristics |
| Pausing | Passages about the importance of taking breaks and how to spend them | Characteristics of the session |
| Activities experts by experience mild-to-borderline disability | What can experts by experience (not peer-mentors) do in treatment? | Role of the peer-mentor |
| Similar/different anxiety or therapy | Patient-peer alignment: whether the patient and peer-mentor should have the same fears or have had the same form of therapy | Characteristics of the peer-mentor |
| Self-efficacy patient | The self-efficacy that a patient may experience during or after treatment | Result of the intervention |
| Cooperation between therapist and peer-mentor | Passages about the collaboration between the therapist and peer-mentor | Relationships |
